# Experiences for Geriatric Care from Nursing Students’ Knowledge: A Qualitative Approach

**DOI:** 10.3390/nursrep14020056

**Published:** 2024-03-27

**Authors:** Elsa Gil-Mateu, Silvia Reverté-Villarroya, Núria Albacar-Riobóo, Josep Barceló-Prats

**Affiliations:** 1 Nursing Department, Campus Terres de l’Ebre, Universitat Rovira Virgili, Avenue Remolins, 13-15, 43500 Tarragona, Spain; elsa.gil@urv.cat (E.G.-M.); nuria.albacar@urv.cat (N.A.-R.); 2 Advanced Nursing Research Group, Universitat Rovira i Virgili, 43002 Tarragona, Spain; josep.barcelo@urv.cat; 3 Nursing Department, Campus Catalunya, Universitat Rovira Virgili, Avenue Catalunya, 35, 43002 Tarragona, Spain

**Keywords:** geriatric, nursing students, practice, qualitative analysis, teaching methods

## Abstract

(1) Background: Studies have shown that clinical experience has an impact on how students perceive geriatric care. The vulnerability of older people particularly allows students to reflect on and evaluate their learning. In this context, communication between tutors and students is important to guiding a contextualized view of the complexity of clinical situations. The principal objective was to explore the feelings, perceptions, and experiences of nursing students in geriatric care units during their practices. (2) Methods: This is a qualitative study using content analysis where the data collected were analyzed deductively. An intentional sample of 81 nursing degree students enrolled in the subject of clinical practices. During these sessions, a dynamic discussion forum was incorporated. (3) Results: There were 6 forums with a total of 591 participants, with an average of 98.5 per forum. Four categories emerged: humanization, geriatric nurse, aging, and learning. (4) Conclusions: A change of management oriented to the person-centered model would improve the quality in the residences and as well as in the expectations of the students towards geriatric nursing. Changing perspectives could be a way to confront and become aware of the fallacies of care that have been evidenced. This study was not registered.

## 1. Introduction

Nursing, as a discipline, is the result of the evolution of the activity of care within contemporary societies, giving a professionalized character to the set of tasks related to care. For this reason, this discipline has needed to develop its own body of scientific knowledge in order to define its nursing activities, to develop their teaching and research, as well as to improve their healthcare practice. The ultimate aim of all these contributions has been to increase the quality of care for the individual, the family, or the community. Consequently, caregiving is a complex practice that requires integrating diverse elements. According to Brykczynska, the essential points of care are compassion, competence, confidence, trust, and awareness. These are basic and inescapable virtues that are required to provide quality care for a human being. They are both personal and professional habits and are mutually necessary [1].

In this idea, training in all the capacities of the human being in order to be able to respond to the problems that life holds becomes the primary goal of education. So, the intersection between attitudes, knowledge, and skills is what they call competence. Acquiring new skills is not only an activity prior to work or what we do in isolation, but is carried out in the course of the work itself, becomes a fundamental piece in its acquisition and development, and consequently, in the process of professionalization. The student is competent because he feels and reflects, and acts accordingly on his knowledge, skills, motives, and values, with flexibility and perseverance in solving problems presented by the practice. And this is what is called reflexive competence, a transversal competence [2,3].

In fact, it is in actual clinical practice—conceived as an open, dynamic, and changing space characterized by the complexity its situations— where the students find a setting conducive to carry out processes of reflection, deliberation, clinical reasoning, and decision-making [4,5,6].

Nursing in the 21st century is capable of addressing care from an ethical and moral standpoint, but reaching this level of maturity requires changes in curricular focus, institutions, and the profession itself. Nursing, therefore, can be considered a dualistic discipline. It is a science of health, but also of the human, trying to distance itself from reductionist tendencies [7]. There are different theories and models that attempt to address this need to find meaning in care.

Certain phenomenological theories propose discovering meaning in these human experiences by directing care towards the patient’s world, which is known as “being with” the person [8,9]. For example, according to Watson, care is a moral idea, rather than a work-oriented attitude. Internalizing and mastering these concepts are what allow care workers to operate in the context of human complexity. Parse’s model presents us with the “Human Becoming Theory” in which, according to the author, “becoming” refers to the possibilities within a human being or what we are in potentiality. For Benner, a person interprets their symptoms in relation to the impact these may have on what is important to them, which includes family, work, leisure, and relationships. Therefore, identifying what is important to the patient is the key to caring for them meaningfully. Benner associates the clinical judgement that determines action with Schön’s practical reasoning, defining it as reasoning in transition, embedded in an interaction with the person and family that cannot be converted into mere procedures or techniques, but arises from an insight into each situation and the knowledge of how to deal with them [9]. According to this author, the basic competencies acquired by students with university training are completed with experience and permanent reflection. The overarching idea in all these models and paradigms is to understand the health–illness spectrum as a life experience that is expressed through narrative that provides nursing with a profound knowledge linking moment, care, person, and situation [10].

In the field of geriatric nursing, the growing demand for care caused by an increasingly ageing population is a challenge for healthcare services. The vulnerability of older people particularly allows students to reflect on and evaluate their learning. Studies have shown that clinical experience has an impact on how students perceive this field [10,11,12].

In this context, communication between tutors and students is important to guiding a contextualized view of the complexity of clinical situations, the analysis and elaboration of clinical reasoning, decision-making, and the exercise of care [13,14]. Teachers are thus expected to use reflective practice, promoting the use of technology, to help students contemplate positive as well as negative experiences in both clinical and academic settings. Present day students belong to a generation that understands information and communication technologies (ICTs) as part of their everyday environment [15,16]. Universities and organizations need to innovate and make constant improvements to adapt curricula to this increasingly virtual environment. As a result, active methodologies such as forums, the flipped-classroom, or conceptual maps are a strategic framework for student-centred learning, steering trainees towards action [16]. However, in the case of clinical training, it is difficult to obtain instruments that allow for an active methodology outside the classroom.

The overall objective of this study was to analyze the experiences that arise from nursing students in their clinical practices in geriatric care units. By encouraging students to go through a reflective process, perceptions and emotions emerge that empower them as agents of transformation within their future areas of work and can support their nursing practice.

## 2. Materials and Methods

### 2.1. Design

This is a qualitative study using a using content analysis [17]; the data collected were analyzed deductively. We recognize that findings are interpretative, and they have been constructed with the participants the of the study. This approach was considered the most appropriate to exploring the reality of students undergoing clinical training in geriatric services, allowing for needs analysis and the implementation of future strategies. An analysis was carried out for quantitative and qualitative variables of the participants and of forum interventions using means, standard deviations (SDs), and percentages.

### 2.2. Participants

A total and intentional population of 81 fourth-year nursing degree students enrolled in clinical practicums in geriatric care units during the academic years 2018–2019 and 2019–2020 were selected.

### 2.3. Data Collection

The data were collected between January 2019 and March 2020. During this period the students carried out their clinical practices. These practices were divided into cycles of 5 weeks each. In total there were 6 cycles of practices, and each cycle was accompanied by a virtual forum. Data were extracted from this forum. (Table 1)

### 2.4. Intervention

Each student chose a different service. During clinical practice, tutors initiated a discussion forum and students were encouraged to participate in it during the course of their internship, the objective of the forum was to maintain a virtual community, where they can express their different experiences. Training was provided to tutors who actively participated in the forum. These tutors served as reference points for the students, motivating them, providing them with information, and addressing any issues that arose while keeping the virtual environment alive. The forums began with a presentation of their practice sites. The tutor responded or encouraged students to bring up topics of interest. The students had to respond with short interventions (4 or 5 lines), answering the tutor’s question and following the students’ thematic thread. At the end of each cycle, a face-to-face seminar was held, where all the information shared and generated in the virtual environment was reviewed [18].

### 2.5. Qualitative Analysis

An in-depth reading of the forums was carried out by S.R.-V. and E.G.-M and the text was coded into units of meaning. These were further coded, compared, contrasted, and ordered into subcategories and finally grouped into main categories representing the main themes of discourse. The data were validated by all study participants. The data were managed using the open-source Weft QDA 1.0.1 for Windows software and analyzed using content analysis [17]. The consolidated COREQ qualitative research reporting criteria were followed [19].

The codes, subcategories, and categories provided the structure of the discourse. In order to further validate the research, the content was analyzed by the students in their end-of-practicum seminars.

### 2.6. Ethics

The study obtained approval from the Pere Virgili Institute for Health Research’s Drug Research Ethics Committee, with the code 121/2020. The introductory workshop of the practicum provided information on the study, the functioning of the forum, and its voluntary participation. All participants signed the corresponding informed consent voluntarily. Non-participation did not entail a change in the treatment or the follow-up to the student. To maximize confidentiality, no names were registered or encoded. The study was carried out in accordance with the precepts of the Declaration of Helsinki [20].

## 3. Results

A total of 81 students participated. Six forums were held with a total of 591 interventions, with an average of 98.5 per forum. In total, 70.6% of the students were women with an average age of 21.18 years (SD = 2.18).

In the data analysis, the following thematic categories emerged: humanization, geriatric nurse, ageing, and learning (Table 2).

### 3.1. Categories

#### 3.1.1. Humanization

Students reflect on humanized care, noting deficiencies in geriatric services. They discussed people-centered care, a practice that needs time, flexibility, knowledge of people, and their emotions, characteristics that they do not observe in clinical practices.

*“Although I believe that patient care is becoming more humane and the ethical basis of care is being taken into account, there is still work to be done to establish a person-centred model of care for the older people.”*

(GG6)

*“These are people who need comprehensive attention and care, centred on the person and not on the illness, and this requires time and personnel that are not available.”*

(GG4)

They discussed the role of the family in the humanized care model, and they talked about changes in family structures and increasing the proportion of older people living alone.

*“I noted that the ageing of the population and changes in family structures have led to an increase in the need for health care resources for older people.”*

(GG2)

*“We judge the family of geriatric patients, and this is a big mistake on our part. Each person has their own story and it is important to know their background in order to understand the situation they are in.”*

(GG3)

Restraints pose an ethical problem for students, presenting them with a theoretical and practical dilemma that is difficult to address.

*“What I have observed in practice is that due to lack of time and staff, they prefer mechanical restraint first, but often even pharmacological restraint is used several times in the same shift. I personally believe that it should be eradicated as far as possible because I think it is inhumane.”*

(GG3)

Finally, communication becomes a therapeutic action inherent in humanized care. Communication skills are recognized as a valuable tool for improving a patient’s prognosis, although there is an increasing lack of communication skills, particularly in the case of people with cognitive impairments and as a result of the mechanization of care.

*“We need to explain what we do and how we do it, but most of the time we assume that patients already know what we are going to do because we have done it more than once or because we think they are not interested or will not understand it.”*

(GG4)

*“Active listening is a practice that we should always adopt and there should be no excuses for not doing it, as it is the basis of communication and of much nursing care.”*

(GG5)

#### 3.1.2. Geriatric Nursing

Certain moments in caregiving are stressful for students as they involve emotional processing and management.

*“And what I wanted to ask is whether you all are also so affected by the unexpected death of a patient you had grown fond of. I think I lack the skills to not carry that home with me, I feel sad on these occasions…”*

(GG5)

They addressed the problems of working conditions offered in geriatric institutions.

*“As nursing students, seeing the state of geriatric care, it discourages us that once we have finished our degree and in the world of work, geriatric centres are one of our last preferences, not because of the type of patients, but because of the type of contracts, the excess workload, the lack of personnel, etc.”*

(GG6)

They were very critical on the issue of clinical safety, whether in medicine preparation, information transfer at shift changes, or in the management of protective isolations.

*“The criteria for protective isolation are not met, we find doors to isolated rooms open, patients with multidrug-resistant bacteria walking down the corridor, isolation wards where one person is infected and another is not.”*

(GG2)

*“Good prevention is key to reducing the volume of care and complications, allowing more attention to be devoted to preventing other conditions, improving care and hygiene. ”*

(GG4)

#### 3.1.3. Ageing

The informants reflected on the social problems of ageing and talk about loneliness among older people.

*“These are people who find themselves in a complicated social situation where they spend many hours alone, without company, which means that they cannot express themselves.”*

(GG4)

They also discussed the stigma around older people, especially highlighting diseases involving cognitive impairment.

*“I have seen stigmatization towards the older people. There are gestures and actions, for example in Alzheimer’s, and they don’t give these the importance they deserve.”*

(GG3)

They also highlighted the apparent frailty of older people, noting that sometimes their own actions are not always correct.

*“It is very important to give the patient autonomy, because we often unintentionally deny it, thinking t‘hey are older, they can’t manage alone’. I tend to think that because I see them as frail.”*

(GG6)

This raised the issue of the institutional mistreatment of older people in residential care settings.

*“We also find a lot of news where there is talk of elder abuse, and they appear to be more vulnerable because of their illnesses.”*

(GG3)

Trainees were also concerned about the amount of medication received by people living in geriatric institutions.

*“The issue of polymedication among the older people is huge. I think that we should try to prioritise and minimise the administration of medication.”*

(GG4)

Finally, they were struck by the issue of death and the finitude of this stage of life.

*“Many of the patients I have had who have been suffering have often commented on wanting to end the situation or have expressed a wish to die.”*

(GG2)

#### 3.1.4. Learning

Students debated the low social value placed on geriatric care as a speciality, although they appreciated the learning opportunity.

*“We all agree that geriatrics is a greatly undervalued speciality, but I think that doesn’t take away from the good moments. In addition to having learned a lot about ulcers and care, I have also been able to see the evolution of the different patients, the way patients and professionals interact.”*

(CG6)

*“As professionals, we have to continue updating the knowledge we already have, as well as expanding it. During this course, I have realised that you can always learn more in any area of care, from techniques to how to manage certain behaviours.”*

(GG3)

All this acquired training enabled students to develop critical thinking, in other words, to reflect on and understand their daily work.

*“I‘ve been deepening my knowledge of wound care, a field that I think is very broad and of real importance for our profession. All this knowledge has allowed me to develop optimal critical thinking, being able to decide for myself and apply previously learned techniques.”*

(GG1)

## 4. Discussion

The informants, like some authors, demonstrated that humanization is a complex process involving all dimensions of a person and ranging from policy to culture, healthcare organization, training of health and social care professionals, and the development of care plans [21,22]. Respect for privacy, a person’s autonomy, management of emotions, spirituality, and adequate communication were all mentioned as important factors in humanizing care.

A person-centred model, therefore, arises, where the aim is to generate home-like environments, focusing on the preferences and needs of older people and on the ability of the professionals involved to learn about these preferences, patients’ life histories, and diversity [23].

The students recognize the necessity of working within this model of care, where the aim is to work with people and not with diseases. They see the need to include the family as part of care, although they note that changes in the social structure of families and carers make this challenging to achieve. Previous studies confirm these structural changes and uphold the role of family and social networks as institutions conducive to active ageing [24,25].

In order to implement this model, existing research emphasizes the need for changes in working conditions and staffing ratios. López et al. [26] concluded that good care is related to both work and personal resources. They also noted that professional strain must be reduced for the promotion of quality care. Institutions do not always respond to the demands of professionals, as demonstrated in the study by Dwyer [27] where nurses felt devalued by the system. The students associate this lack of personnel with the mechanical and pharmacological restraint of residents, emphasising the need to eradicate this practice. According to them, and in line with the literature [28,29], health can be said to improve when patient care is based on a person-centred, non-restraint model.

Delving into reflective thinking when students were in their clinical practices allowed them to reflect on the role of institutions in working on the people-centered model. This model has been called for a long time in care models, so the paradigm of transformation defends a more humanized practice, identifying what is meaningful for the person. Students identify structural factors that make it difficult to work along these lines.

Thus, although the current demand for geriatric nurses is well documented, mechanized care, clinical safety concerns, and the professional burnout highlighted make students consider this speciality among their last professional preferences. This attitude, which is also evidenced in the literature [30,31], indicates that a change in the management model is vital to improving the quality of care. Other studies [32,33] have shown that this attitude is also related to the emotions associated with ageing. This is reflected in the students ‘conversations where the end of life and close relationships with the residents can provoke sadness.

This encounter with ageing makes them reflect on issues such as frailty, polymedication, and the overall complexity of caring for the older people. These results are consistent with studies [21,34] showing that although geriatrics is a speciality that recognizes social dimensions and claims to promote a holistic approach, the training of professionals is focused on pharmaceutical interventions, and more centred on cure than on care, despite professionals themselves recognizing that the best treatments are not pharmacological.

This leads students to consider elder abuse, which Leibing [21] defines as structural violence. Previous authors [26,35,36,37,38,39] provide reasons why the older people are more vulnerable to abuse.

Students reflect on the vulnerability of the older people; they say they have difficulty addressing issues such as the end of life. Studies by Reverté et al. or Osorio et al. conclude that these situations create anxiety [40,41]. Consequently, the results of this study underline the need to promote attitudes aimed at alleviating this anxiety about ageing and to educate students in good emotional management to cope with the end of life. Some authors [42,43,44] have pointed out that support integrated into clinical practice along with critical reflection improve students’ knowledge, understanding, and attitudes towards older people [45,46,47,48].

The clinical practices are the moment when the student arrives at a professional situation and faces an intense life experience, related to the illness, pain, suffering, and death of patients. These experiences impact their personality. The implementation of an easily accessible virtual space without time restrictions, in small groups, where lived situations can be shared and exposed, accompanies them in their learning and helps them to develop the reflective thinking and reduce the anxiety described in some studies [4,49].

The need to be critical and reflective is considered one of the challenges and solutions for teaching–learning processes in clinical practice environments. Active methodologies, such as forums, are a resource for exchanging knowledge among participants; they are educational strategies that allow the development of critical thinking in the practicums, where the tutor and the students need virtual spaces to dialogue and seek ethical, competent, and reflective actions [18].

Finally, forums offer a method for accompanying clinical practice with a space for sharing experiences. It is a space to contribute, with a deep reflection, about the experiences, as the described categories show. As we have seen, this type of tool allows students to deal with complex issues that arise from the training, to improve their experience, and thus to promote and maintain an interest in geriatric nursing.

## 5. Study Limitations

The study was aimed at senior nursing students. The study sample was intentionally collected, and although participation was voluntary and the study was reported, this could have limited the results. Also, the fact that the female sex predominates is a factor that could have influenced the characteristics of the participations.

## 6. Conclusions

This study demonstrates that changes in institutional practice towards a more person-centred model of care would both improve the quality of nursing homes as well as the expectations of students towards geriatric nursing as a professional option. Changing perspectives would raise awareness and help address the shortcomings in care that students have brought to light. This requires nursing practice to engage in a process of reflection to integrate people’s beliefs and values, apply knowledge and clinical judgement, organize resources, and evaluate the quality of its interventions.

This study has made it possible to explore trainees’ feelings and perceptions on caring for older people, so that teachers can understand their attitudes and concerns and address any misconceptions. This approach has allowed students to reflect on and understand the experience of professional clinical practice. Their experiences have brought up different feelings regarding their inability to look after a patient’s best interests. Understanding how and why such emotions arise can help to improve nursing practice, particularly in geriatric nursing, and to ultimately improve care for older people, their families and their communities.

## Figures and Tables

**Table 1:** Virtual forum codes.

Forums	Code	Number of Students (*n* = 81)
January/February 2019	GG*1	12
February–March 2019	GG2	15
March–April 2019	GG3	14
April–May 2019	GG4	12
January–February 2020	GG5	14
February–March 2020	GC6	14

*GG = Geriatrics Group.

**Table 2:** Qualitative Analysis.

Category	Definition	Subcategories
Humanization	To value the dignity and individuality of the person, based on a comprehensive approach where the biological, psychological, social, and spiritual dimensions interact.	Person-Centered Care Family Restraints Communication
Geriatric Nursing	Ability to put into practice the knowledge, skills, and attitudes of the nursing profession at the service of the resolution and prevention of a health problem.	Emotion management Geriatric Institutions Clinical safety
Ageing	The set of morphological, functional, and psychological changes that entail changes in the structure and function of the different systems, increasing the individual’s vulnerability to environmental stress and disease.	Loneliness Stigma Fragility Abuse Finitude Polymedication
Learning	The process of acquiring knowledge, skills, values and attitudes through study, teaching, or experience.	Training Critical thinking

## Data Availability

The data presented in this study are available on request from the corresponding author.

## Public Involvement Statement

Final year nursing degree students participated in this study.

## Guidelines and Standards Statement

This manuscript was drafted against the Strengthening the Reporting of Observational Studies in Epidemiology (STROBE) and the guidelines were followed in this observational study.
